# CRISPR/Cas9-Mediated Foxp1 Silencing Restores Immune Surveillance in an Immunocompetent A20 Lymphoma Model

**DOI:** 10.3389/fonc.2020.00448

**Published:** 2020-04-03

**Authors:** Suet Ling Felce, Amanda P. Anderson, Shaun Maguire, Duncan M. Gascoyne, Richard N. Armstrong, Kah Keng Wong, Demin Li, Alison H. Banham

**Affiliations:** ^1^NDCLS, Radcliffe Department of Medicine, John Radcliffe Hospital, University of Oxford, Oxford, United Kingdom; ^2^Nuffield Department of Orthopaedics, Rheumatology and Musculoskeletal Sciences, University of Oxford, Oxford, United Kingdom; ^3^Genetics and Genome Biology Program, Haematology Section, Division of Haematology/Oncology, Department of Paediatrics, The Hospital for Sick Children, Toronto, ON, Canada; ^4^The Marrow Failure and Myelodysplasia Program, Haematology Section, Division of Haematology/Oncology, Department of Paediatrics, The Hospital for Sick Children, Toronto, ON, Canada; ^5^Department of Immunology, School of Medical Sciences, Health, Universiti Sains Malaysia, Kota Bharu, Malaysia

**Keywords:** lymphoma, MHC class II, diffuse large B-cell lymphoma, FOXP1, immune response

## Abstract

The interaction of lymphoma cells with their microenvironment has an important role in disease pathogenesis and is being actively pursued therapeutically using immunomodulatory drugs, including immune checkpoint inhibitors. Diffuse large B-cell lymphoma (DLBCL) is an aggressive high-grade disease that remains incurable in ~40% of patients treated with R-CHOP immunochemotherapy. The FOXP1 transcription factor is abundantly expressed in such high-risk DLBCL and we recently identified its regulation of immune response signatures, in particular, its suppression of the cell surface expression of major histocompatibility class II (MHC-II), which has a critical role in antigen presentation to T cells. Using CRISPR/Cas9 genome editing we have depleted Foxp1 expression in the aggressive murine A20 lymphoma cell line. When grown in BALB/c mice, this cell line provides a high-fidelity immunocompetent disseminated lymphoma model that displays many characteristics of human DLBCL. Transient Foxp1-depletion using siRNA, and stable depletion using CRISPR (generated by independently targeting *Foxp1* exon six or seven) upregulated cell surface I-A^b^ (MHC-II) expression without impairing cell viability *in vitro*. RNA sequencing of Foxp1-depleted A20 clones identified commonly deregulated genes, such as the B-cell marker *Cd19*, and hallmark DLBCL signatures such as MYC-targets and oxidative phosphorylation. Immunocompetent animals bearing Foxp1-depleted A20 lymphomas showed significantly-improved survival, and 20% did not develop tumors; consistent with modulating immune surveillance, this was not observed in immunodeficient NOD SCIDγ mice. The A20 Foxp1 CRISPR model will help to further characterize the contribution of Foxp1 to lymphoma immune evasion and the potential for Foxp1 targeting to synergize with other immunotherapies.

## Introduction

The tumor microenvironment plays a critical role in lymphoma pathogenesis, with immunomodulatory and immune evasion strategies facilitating tumor growth (1). Immunotherapy is a rapidly growing area of translational research in this field, and immune checkpoint inhibitors that activate effector T-cell function by blocking the PD-1/PD-L1 and CTLA-4/B7 pathways to reactivate the activity of effector T cells have already shown promise in hematological malignancies (2). However, these treatments come with a substantial financial cost and adverse side effects, and it remains challenging to identify those subgroups of patients who will respond effectively. Importantly, understanding the interaction of lymphoma cells with their microenvironment offers the opportunity to determine the most effective available treatment options, and the possibility of identifying new avenues for therapeutic intervention.

Diffuse large B-cell lymphoma (DLBCL) is the most common subtype of non-Hodgkin's lymphoma. The germinal center B-cell-like subtype (GCB-DLBCL) responds well to multi-agent R-CHOP (rituximab–cyclophosphamide, doxorubicin, vincristine, and prednisone) immunochemotherapy. However, the aggressive activated B-cell-like subtype (ABC-DLBCL) has only a 45% 3-year survival, and most patients relapse with refractory disease (3, 4). Stratification of DLBCL patients and new molecularly-guided therapies are urgently needed to improve the limited treatment options available for ABC-DLBCL, and these are actively being developed (5).

Loss of major histocompatibility class II (MHC-II) expression is a devastating determinant of poor outcome in DLBCL patients, regardless of other prognostic factors (6, 7). This loss of MHC-II expression is most commonly observed in the high-risk ABC-DLBCL subtype (8, 9) and R-CHOP treated DLBCL patients with reduced HLA-DRA expression exhibited significantly inferior survival (9). Reduced MHC-II expression impairs antigen presentation and therefore represents a direct mechanism by which tumor cells escape host immune surveillance. High-level expression of the FOXP1 fork head transcription factor is associated with the ABC-DLBCL subtype and has been correlated with a poor response to CHOP and R-CHOP therapy in multiple studies (10). Gene expression profiling studies have shown that FOXP1 drives multiple oncogenic pathways and, importantly, that it is a transcriptional repressor of MHC-II expression in DLBCL (3, 9).

We previously proposed that targeting the FOXP1 pathway offered an opportunity to restore antigen presentation and immune surveillance in high-risk DLBCL patients (9). To experimentally test this hypothesis we have used CRISPR/Cas9 genome editing to deplete Foxp1 from an immune competent lymphoma model. We selected the aggressive murine A20 model of B-cell lymphoma, which mimics key aspects of human DLBCL and has previously been used to study the anti-lymphoma immune response and test therapeutic approaches in an immunocompetent host (11–13). Notably, ectopic expression of allogeneic MHC-II in A20 lymphoma cells has already been shown to enhance T-cell proliferation (14). Furthermore, we previously demonstrated that the expression of multiple *Foxp1* transcripts with alternate 5′ exons and full-length and smaller Foxp1 proteins, comparable to those observed in human ABC-DLBCL cell lines, are conserved in the A20 lymphoma model (15). Here, we show that Foxp1 silencing increases cell surface MHC-II (I-A^b^ in BALB/c mice) expression, and significantly impairs A20 lymphoma growth *in vivo* in an immune-competent but not an immunocompromised host. This A20 Foxp1 CRISPR model should prove valuable for studying the contribution of Foxp1 to immune evasion in aggressive lymphoma.

## Materials and Methods

### Cell Culture

The A20 mouse B-cell lymphoma cell line was obtained from ATCC and maintained in RPMI supplemented with 10% fetal bovine serum, penicillin/streptomycin, and 50 μM β-mercaptoethanol, in a 37^o^C 5% CO_2_ humidified incubator. Additional human DLBCL cell lines were sourced and maintained as described previously (16).

### Transient *Foxp1* Silencing

*Foxp1* expression was silenced in A20 cells by electroporation using program L-016 in an Amaxa® Nucleofector® IIb Device (Lonza, Slough, UK). Briefly, 2 × 10^6^ cells were electroporated in Solution V supplemented with 2 μM Foxp1-targeting MSS246912 (si*FOXP1* #1) or MSS246913 (si*FOXP1* #2) Stealth RNAi™ (Thermo Fisher Scientific, Paisley, UK), or negative control siRNA duplex (Stealth RNAi^TM^ Low GC, Thermo Fisher Scientific), and harvested after 48 h for flow cytometry, Western blotting and qPCR analysis. Supplementary Table 1 provides the siRNA sequences.

### Protein Assays

Nuclear proteins were isolated from cells for Western blotting using the Panomics Nuclear Extraction Kit (Affymetrix, Thermo Fisher Scientific). Whole cell lysis was carried out using Mammalian Protein Extraction Reagent (Thermo Scientific). Protein concentrations were determined using the Pierce BCA assay kit (Thermo Fisher Scientific), before loading 15 μg onto a pre-cast SDS-PAGE gradient gel (NuPAGE® Novex® 4–12% Bis-Tris Protein Gels, Life Technologies, Thermo Fisher Scientific). Proteins were transferred onto a nitrocellulose membrane (Amersham™ Protran™ NC membrane, GE Healthcare, Chalfont St Giles, UK) by conventional semidry blotting, before incubating with antibodies (described in Supplementary Table 2). Detection of β-actin or Nucleophosmin (Npm) expression was used to control for sample loading. Blots were incubated with Amersham™ ECLPrime Western Blotting Detection Reagent (GE Healthcare) and visualized using Syngene G:BOX Chemi XRQ (Syngene, Cambridge, UK) by chemiluminescent detection or by exposure to X-ray film (Scientific Laboratory Supplies, Nottingham, UK).

### Flow Cytometry

Cells were incubated with mouse-specific antibodies recognizing MHC-II, Cd19 (both eBioscience, Thermo Fisher Scientific), or Cd74 (R&D Systems, Abingdon, UK) conjugated to fluorophores (Supplementary Table 2). After a PBS wash and 1% formaldehyde fixation, flow cytometric analysis was performed using a FACSCalibur (Becton Dickinson, Wokingham, UK) and data were analyzed using FlowJo software (Becton Dickinson).

### Quantification of Gene Expression by qPCR

RNA was extracted using Qiagen's RNeasy® Mini Kit and 1μg was reverse transcribed using Superscript® III reverse transcriptase, according to the manufacturer's instructions (Invitrogen, Thermo Fisher Scientific). Complementary DNA (cDNA) was diluted 1/5 and subjected to PCR using the SYBR® GreenER™ qPCR Supermix (Invitrogen) on a Biorad MJ Chromo4 thermal cycler (see Supplementary Table 3 for primer sequences). The cycle threshold (Ct) was determined for each sample, and target gene Ct-values calculated (ΔCt = Ct *control sample*- Ct *test sample*). For qPCR using FAM dye-labeled Taqman probes (Supplementary Table 4), target gene Ct-values were normalized by subtracting that of an endogenous housekeeping gene (VIC dye-labeled; ΔCt = Ct FAM – Ct VIC). The expression of gene-specific mRNA was normalized using *18S, B2m*, or *Hprt1* and calculated by subtracting the normalized Ct-values obtained from the control sample to determine relative expression (2^ΔΔ*Ct*^).

### CRISPR/Cas9 Knockdown of Foxp1 Expression

Guide RNA (gRNA) sequences targeting murine *Foxp1* exons 5, 6, and 7 (denoted as E5, E6, and E7, respectively—shown in Supplementary Table 5) were designed using an online tool (http://crispr.mit.edu/) in order to minimize off-target effects, and to precede a protospacer-adjacent motif (PAM). Potential CRISPR off-target genes were identified using BLAST, and genomic regions were checked for mutations by viewing data files from subsequent RNA sequencing in a genome browser. Genes with the highest sequence matches to the *Foxp1* CRISPR guides targeting E6 (*Map1b, Ovgp1*) and E7 (*Zfp804b, Rhpn2, Tmem194*) also did not show altered transcript expression in RNA sequencing analysis. Custom oligonucleotides encoding sense and antisense guide sequences (Sigma-Aldrich, Poole, UK) were phosphorylated using T4 polynucleotide kinase (New England Biolabs, Hitchin, UK) for 1h at 37°C, and annealed following the manufacturer's protocol. The pX330-U6-Chimeric_BB-CBh-hSpCas9 was a gift from Feng Zhang (Addgene plasmid # 42230; http://n2t.net/addgene:42230; RRID: Addgene_42230). It encodes both the expression cassette required for the production of gRNA sequences and theCas9 enzyme, and was digested overnight at 37°C with restriction enzyme *BbsI* (NEB). In accordance with the Feng Zhang laboratory protocol (17), the linearized vector and annealed oligos were ligated and transformed into *Escherichia coli* Stbl3 competent cells (ThermoFisher Scientific). Subsequent colonies were screened by PCR (see Supplementary Table 6 for primer sequences) and sequences were confirmed by DNA sequencing (Source BioScience Sequencing, Nottingham, UK).

Plasmids encoding the confirmed guide sequences were electroporated into A20 cells using the Nucleofector IIb program described above. After 48 h, transfected cells were sorted for GFP expression by FACS (fluorescence activated cell sorting) and underwent clonal selection using single cell dilution. Clones were screened by Western blotting to confirm Foxp1 knock-down and genome sequencing to map the *Foxp1*mutation.

### Cell Viability Assays

A20 parental and A20 CRISPR-modified cells were seeded at 1 × 10^6^ cells/well in a 6-well plate and incubated for 72 h at 37°C. Cell viability was determined using the trypan blue exclusion test (Sigma-Aldrich).

### Immunohistochemistry (IHC)

IHC labeling of murine tumor tissues was performed after dewaxing and heat-mediated antigen retrieval in 50 mM Tris/2 mM EDTA pH 9.0. Foxp1 expression was detected using a rabbit anti-mouse Foxp1 antibody (D35D10, Cell Signaling Technology, London, UK) and the Dako REAL™ EnVision Detection System (HRP, Rabbit/Mouse), according to the manufacturer's protocol (Agilent Technologies, Stockport, UK). Images were captured using an Olympus BX51 microscope coupled to an Olympus DP70 camera.

### Animal Studies

Female 6- to 8-week old mice (immunocompetent BALB/cAnNCrl and immunocompromised NOD-SCID-gamma JAX strain) were purchased from Charles River Laboratories. Mice were housed in temperature-controlled conditions and had access to sterile water and formulated diets, in individually-ventilated cages. All animal experiments were performed in accordance with the terms of the UK Home Office guidelines and with the approval of the Medical Sciences Animal Ethics Committee, University of Oxford under Project License PPL: 30/3133.

### A20 Lymphoma Tumor Mouse Model

Mice were divided into three groups of 10 (each group receiving parental A20 cells, A20 Foxp1 E6, or A20 Foxp1 E7-targeted cells), anesthetized using isofluorane, and A20 cells (2 × 10^5^) were injected subcutaneously. Mice were monitored for any adverse welfare indications and palpable tumors were measured every 2–3 days using digital calipers. Mice were culled once a tumor GMD (gross mean diameter) of 15 [calculated by (height × width × length)^1/3^] had been reached, or if tumors began to ulcerate. Tumors were removed post mortem for subsequent studies.

### RNA Sequencing

Total RNA was isolated from triplicate A20 parental and A20 CRISPR modified clones using RNeasy® Mini Kit (Qiagen, Manchester, UK). Samples were prepared for RNA sequencing (RNA seq) at the Wellcome Trust Centre for Human Genetics, Oxford, using HiSeq4000. Differentially-expressed genes were determined using edgeR (18, 19) ±1-fold change and FDR adjusted *p*-value *p* < 0.05. Gene set enrichment analysis was performed for all differentially expressed genes using EGSEA (20), and gene sets used were obtained from the Molecular Signatures Database (H, hallmark gene sets; available at http://software.broadinstitute.org/gsea/msigdb/genesets.jsp?collection=H) (21). Pathways which showed opposing directions of regulation, when comparing the E6 and E7 A20 clones (for example upregulation in E7 clones and downregulation in E6 clones), were designated as neutral. RNA-Seq data has been deposited in the Gene Expression Omnibus (GEO) database under accession number GSE139536.

### Statistical Analysis

Experimental data are presented as mean ± standard deviation. For comparison between two groups, statistical analyses were carried out using two-tailed Student's *t*-tests (GraphPad Prism). The Kaplan–Meier method was used for survival analyses, which were compared using log-rank test. A two-sided *p*-value of < 0.05 determined statistical significance in all analyses. Statistical significance levels are denoted as follows: ns, not significant, ^*^p < 0.05, ^**^p < 0.01, ^***^p < 0.001, ^****^p < 0.0001.

## Results

### Transient Foxp1 Silencing in the A20 Model

**Figure 1:**
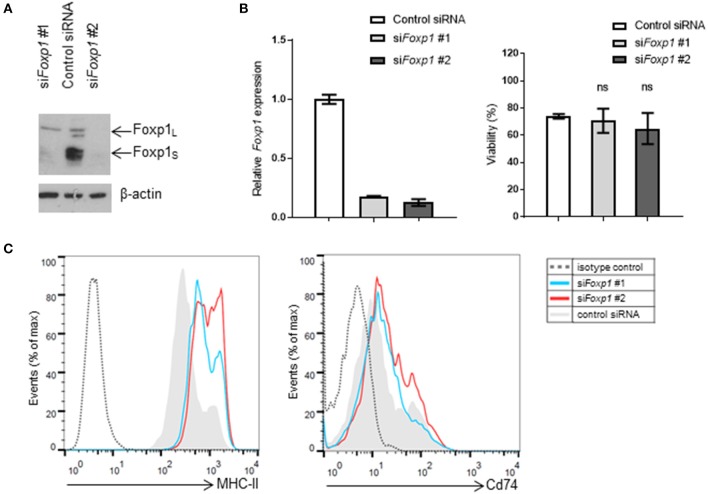
Foxp1 silencing in murine A20 lymphoma cells restores MHC class II expression without impairing cell viability. **(A)** *Foxp1*-targeting siRNA transiently transfected into A20 cells reduced Foxp1 protein and **(B)** *Foxp1*transcript expression, compared to transfection with a non-targeting siRNA control. **(B)** Foxp1 knockdown did not affect cell viability. **(C)** Expression of cell surface MHC-II was increased following Foxp1 knockdown, while only modest upregulation of Cd74 was observed.

Transient *Foxp1* depletion, using two independent siRNAs, was initially used to investigate whether MHC-II (I-A^b^) expression was regulated by Foxp1 in the murine A20 lymphoma model. Both siRNAs silenced Foxp1 expression effectively at the transcript and protein level (Figure 1A), without any adverse effect on A20 cell viability (Figure 1B). MHC-II expression was upregulated on the cell surface after *Foxp1* siRNA, while there was little effect on Cd74 expression (Figure 1C).

### Stable Foxp1 Depletion in the A20 Cell Line Using CRISPR/Cas9 Genome Editing

**Figure 2:**
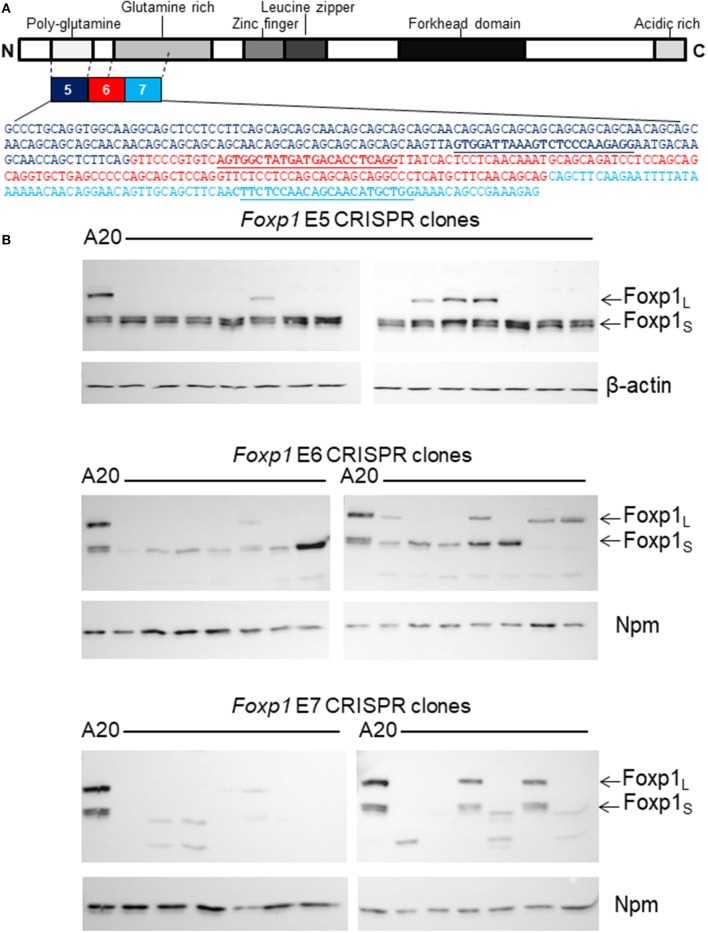
CRISPR/Cas9 genome editing genetically depleted Foxp1in A20 cells. **(A)** Exons 5, 6, and 7 of *Foxp1* were selected for CRISPR/Cas9 targeting. gRNA sequences are shown in bold and underlined. **(B)** Individual CRISPR clones were grown from sorted A20 cells after CRISPR/Cas9 targeting. Most clones showed loss of the full length Foxp1 protein (Foxp1_L_) by Western blotting. Exon 5 targeting did not deplete the Foxp1 short (Foxp1_S_ proteins). Exon 6 targeting reduced expression of the higher molecular weight Foxp1 Foxp1_S_ isoform and several Exon 7 targeted clones no longer expressed any Foxp1. Nucleophosmin (Npm) or β-actin was used as a sample loading control.

As Foxp1 depletion did not impair the viability of A20 cells, a targeted CRISPR/Cas9 genome editing strategy was employed to stably silence *Foxp1* expression. A strategy was designed to disrupt the expression of both the full-length and short isoforms of the murine Foxp1 protein, as they have been reported to share oncogenic and transcriptional activity in human B cells (22). Transcription from alternate 5′ internal promoters generates the smaller FOXP1 proteins in human DLBCL with translation commonly starting in exon 8 (corresponding to exon 6 in the murine *Foxp1* ortholog). Thus, gRNAs were designed to target murine *Foxp1* exon 6 and its flanking exons 5 and 7 (Figure 2A). Panels of Foxp1-targeted CRISPR clones were expanded from individually sorted A20 cells and validated for Foxp1-depletion. Western blotting confirmed that targeting all three exons (5, 6, and 7) depleted the full-length Foxp1 protein in the majority of the clones (Figure 2B). Exon 5 targeting maintained or modestly increased expression of the Foxp1_S_ proteins in the majority of the clones. Exon 6 targeting depleted the higher molecular weight smaller Foxp1 isoform (Foxp1_S_) and in one clone enhanced expression of a Foxp1_S_ isoform, while exon 7 targeting depleted all the Foxp1 isoforms normally present in A20 cells. Several of the CRISPR clones expressed smaller Foxp1 proteins than were typically present in the parental A20 cell line. These are unlikely to be derived from frame shifts causing truncating mutations, as the antibodies used recognize the C-terminus of the Foxp1 protein.

### *In vitro* Characterization of A20 Foxp1 CRISPR Clones

**Figure 3:**
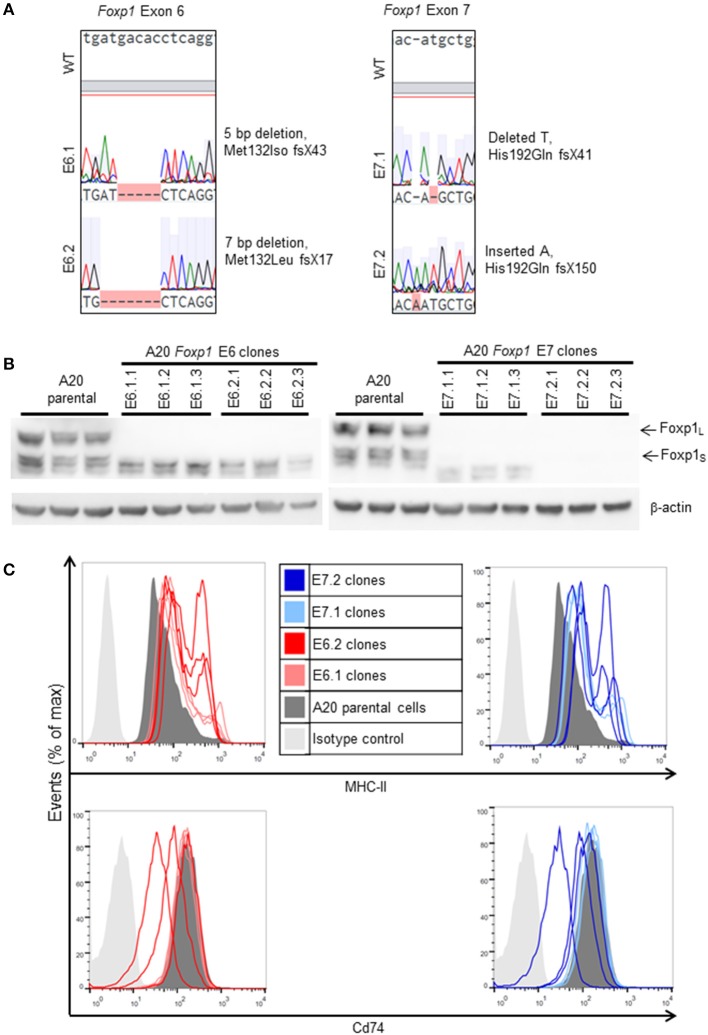
Characterization of A20 CRISPR/Cas9 Foxp1 knockout clones. **(A)** Exon coding DNA from selected clones (E6.1: A20 E6 clone 1; E6.2: A20 E6 clone 2; E7.1: A20 E7 clone 1; E7.2: A20 E7 clone 2) was analyzed by Sanger sequencing and compared to wild-type (WT) *Foxp1*. Each clone contained a deletion or insertion leading to a frameshift and the introduction of a premature stop codon. **(B)** Western blotting of biological replicates, used for RNA sequencing analysis, showed that knockout of Foxp1 protein expression was stable during subsequent passages of each clone. **(C)** MHC-II expression was elevated in all of the clones, while Cd74 expression was unaltered or inconsistently reduced, compared to A20 parental cells.

**Figure 4:**
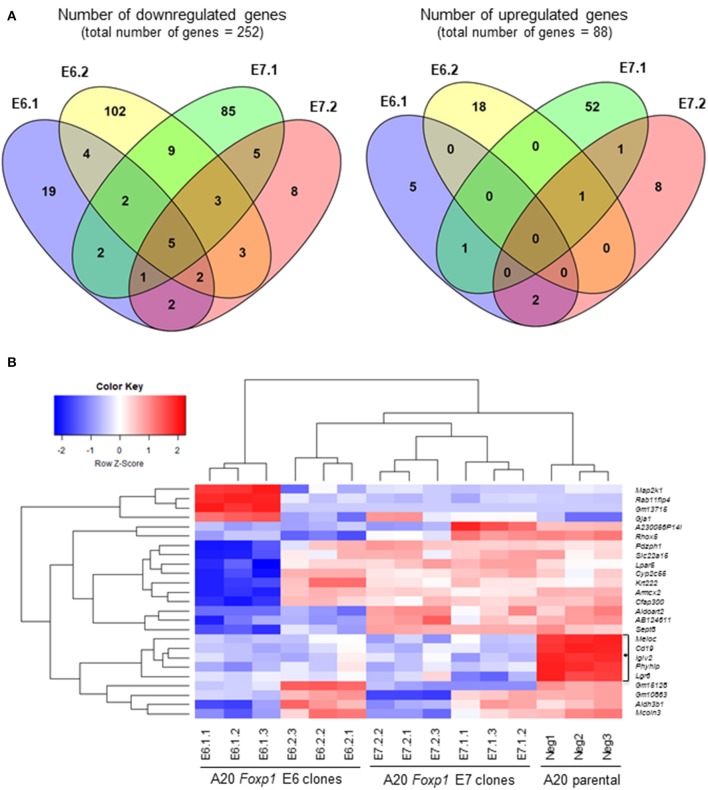
Foxp1 depletion in CRISPR/Cas9 clones alters gene expression and signatures involved in human DLBCL pathogenesis. **(A)** Analysis of differential gene expression was performed after RNA sequencing, comparing the gene expression profile of each clone with that of the A20 parental cell line. Venn diagrams show numbers of genes down or upregulated following Foxp1 knockout (two-fold cut off, FDR adjusted *p*-value, *p* < 0.05). The number of individual genes downregulated in each clone is E6.1 (*n* = 37), E6.2 (*n* = 130), E7.1 (*n* = 112), E7.2 (*n* = 29). The number of individual genes upregulated in each clone is E6.1 (*n* = 8), E6.2 (*n* = 19), E7.1 (*n* = 55), E7.2 (*n* = 12). Gene lists produced from this analysis are provided in Supplementary Table 7. **(B)** Heat map representation of top 25 significantly (*p* < 0.05) differentially expressed genes in Foxp1 CRISPR/Cas9 edited clones compared to A20 parental cells. The genes commonly downregulated in all the Foxp1 CRISPR clones are indicated by a bracket and asterisk. Unsupervised clustering by complete-linkage Euclidean distance was used to generate the heat map dendrograms.

**Figure 5:**
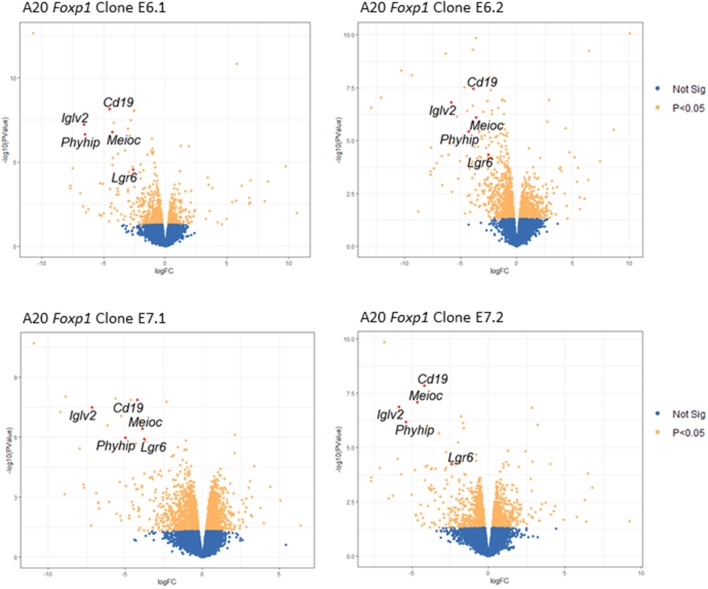
Volcano plots showing changes in gene expression across all four Foxp1-depleted A20 clones. Key genes with significantly (FDR adjusted *p*-value, *p* < 0.05) altered expression in all four A20 clones are shown in red.

Two *Foxp1* exon 6 and two exon 7 A20 CRISPR clones, each with independent genetic changes (Figure 3A) that additionally introduced a premature translational stop codon, were selected for further characterization. Western blotting from three biological replicates, used subsequently for RNA sequencing analysis, confirmed that their Foxp1 protein expression patterns were stable over time (Figure 3B), as was upregulation of cell surface MHC-II protein expression (Figure 3C). RNA sequencing analysis demonstrated deregulated gene expression in the Foxp1-depleted A20 CRISPR clones (Figure 4A), including five genes that were significantly downregulated in all four clones (*p* < 0.05, Figures 4B, 5). There were fewer genes that were significantly upregulated by Foxp1 depletion (88 upregulated genes vs. 252 downregulated genes), and none were common to all four A20 clones.

**Figure 6:**
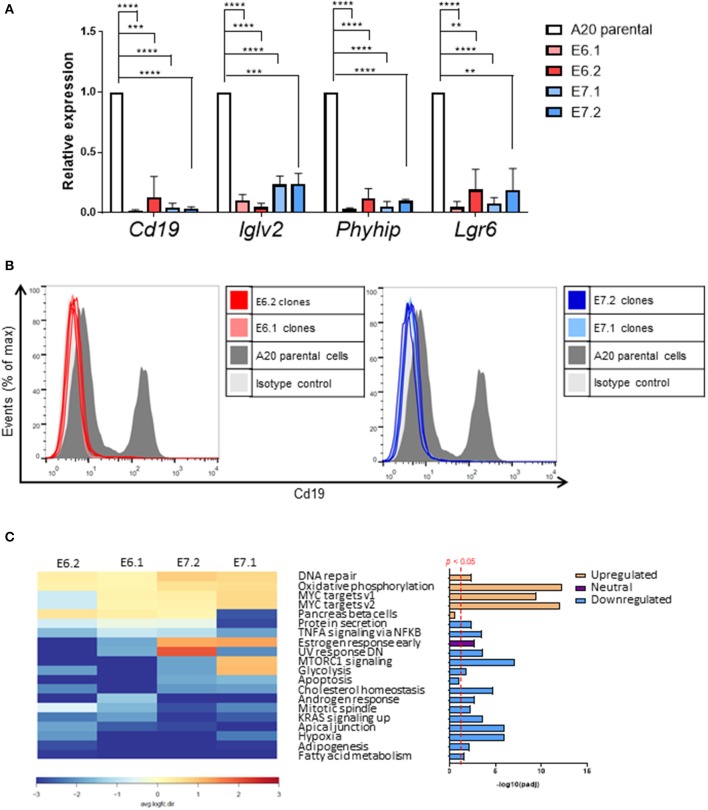
Individual genes most affected by Foxp1 knockout include the B-cell marker Cd19. **(A)** Quantitative PCR independently validates reduced gene expression of *Cd19, Iglv2*, and *Phyhip* and *Lgr6* in Foxp1 depleted A20 clones. **p < 0.005, ***p < 0.001, ****p < 0.0001. Gene expression for each clone was compared with that of the A20 parental cell line using Student's *t*-test. **(B)** Flow cytometry analysis confirms that Cd19 protein expression at the cell surface expression is reduced in Foxp1 knockout cells. **(C)** Gene set enrichment analysis shows that genes in specific hallmark signatures involving Myc and oxidative phosphorylation are affected by Foxp1 depletion. The red vertical line on the lower right panel indicates FDR adjusted *p*-value *p* < 0.05. The estrogen response early pathway is labeled as having a neutral direction of regulation because it is upregulated in the E7 A20 clones and downregulated in the E6 A20 clones.

Quantitative PCR analysis independently confirmed that *Cd19, IgIv2, Phyhip*, and *Lgr6* were significantly downregulated in all four Foxp1 A20 CRISPR clones (Figure 6A). Lgr6 is a G protein-coupled receptor (GPCR) that has been implicated in cancer progression and in activation of Wnt signaling (23). Interestingly, three genes, *Cd19, Lglv2*, and *Phyhip*, encode proteins with an immunoglobulin-like fold or domain that occurs in many diverse proteins in addition to immunoglobulin molecules. The downregulation of Cd19, an important B-cell marker that regulates B-cell expansion and humoral immunity, was confirmed at the protein level on the cell surface (Figure 6B).

Gene set enrichment analysis (GSEA) using the small number of significantly regulated genes (*n* = 340, *p* < 0.05) did not identify pathways common to all four Foxp1 depleted A20 CRISPR clones. Therefore, we used Ensemble of Gene Set Enrichment Analyses (EGSEA), which combines multiple gene set enrichment methods to produce a new ranking that can be more biologically meaningful than results from individual methods and has been designed to analyze murine RNA-Seq datasets (24). EGSEA analysis of all differentially expressed genes identified upregulation of hallmark signatures such as MYC targets and oxidative phosphorylation as the most significantly affected pathways common to our A20 CRISPR clones after Foxp1 depletion (Figure 6C).

### *Foxp1* Depletion Impairs A20 Lymphoma Growth *in vivo*

**Figure 7:**
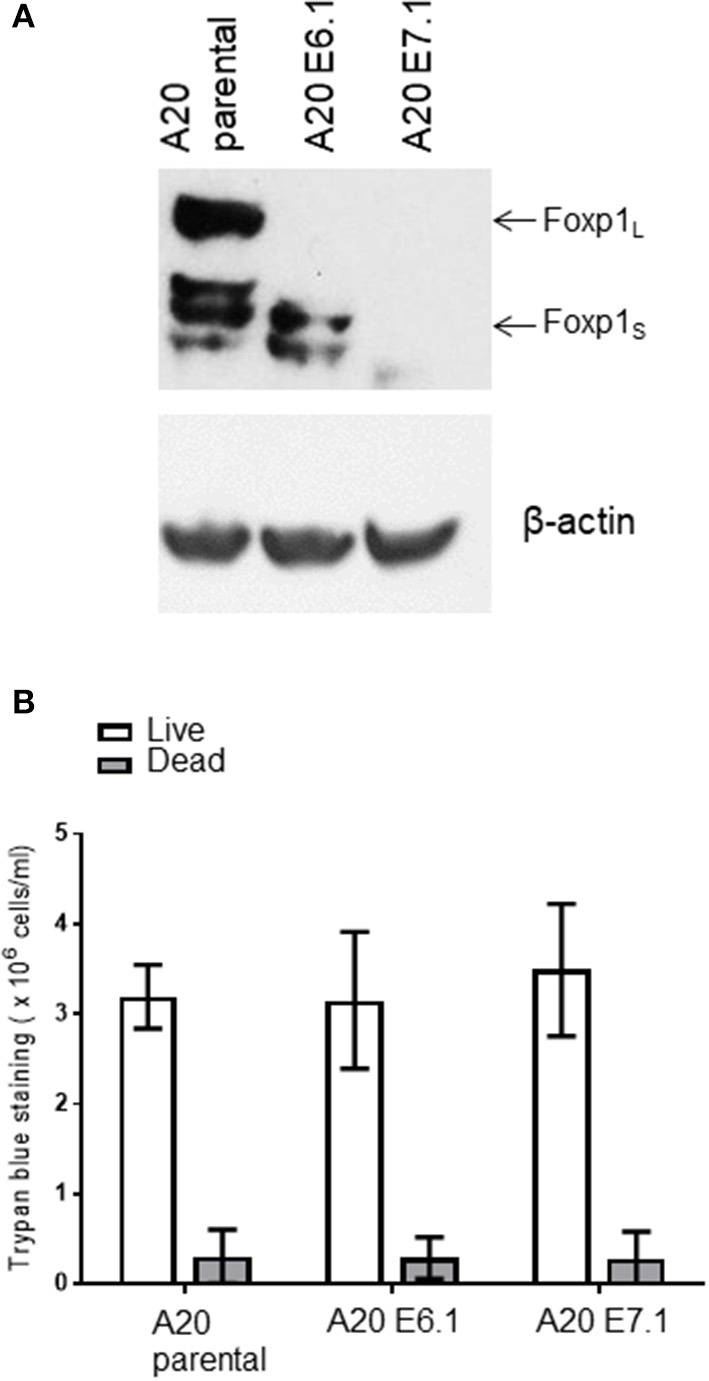
A20 clones used for *in vivo* studies. **(A)** Foxp1 knockout clones prior to *in vivo* inoculation do not show Foxp1 protein expression compared to A20 parental cells. **(B)** Foxp1 CRISPR depletion does not affect cell viability *in vitro*.

**Figure 8:**
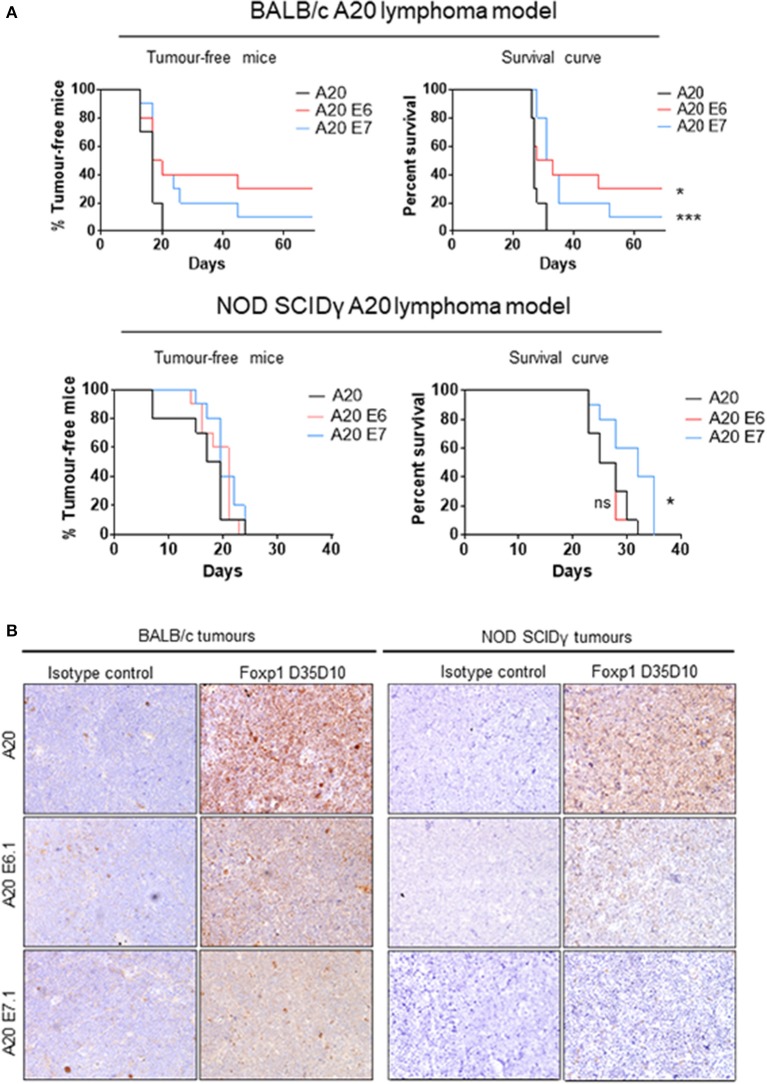
Foxp1 depletion impairs A20 lymphoma growth in a syngeneic immunocompetent mouse model. **(A)** Subcutaneous tumor growth of Foxp1-depleted A20 cells was impaired in immunocompetent BALB/c mice compared with A20 parental cells, and animals with Foxp1-depleted tumors exhibited significantly better overall survival. *p < 0.05, ***p < 0.001. In contrast, Foxp1 knockout cells formed tumors at a similar growth rate to A20 parental cells in NOD SCIDγ immune-deficient mice and only *Foxp1* exon 7 targeting showed a modest improvement in the overall survival of the animals. *p < 0.05; ns, not significant. **(B)** Foxp1-depleted A20 tumors excised from immunocompetent and immunodeficient mice were confirmed to lack tumor cell Foxp1 protein expression by immunohistochemistry.

*Foxp1* exon 6 and exon 7 targeting in A20 both upregulated MHC-II expression and resulted in common patterns of altered gene expression *in vitro*. To exclude the possibility that an *in vivo* phenotype could potentially derive from off-target genome editing, A20 clones generated with independent CRISPR guides targeting *Foxp1* exon 6 or 7 were used for *in vivo* studies. The Foxp1 E6.1 and E7.1 A20 CRISPR clones, and the parental A20 cell line, were implanted subcutaneously in groups of 10 immunocompetent BALB/c mice. The Foxp1 expression level in the cells used for *in vivo* studies and their viability was consistent with previous observations (Figure 7). All of the animals injected with the parental A20 cells had developed palpable tumors by day 20, at which time only 40% of the animals inoculated with the Foxp1 CRISPR clones had palpable tumors (Figure 8A, upper left panel). Indeed three mice in the A20 Foxp1 E6 group and one mouse in the A20 Foxp1 E7 (20%) failed to develop tumors during this experiment. The overall survival of animals with the Foxp1 CRISPR clones was significantly better than those with the parental A20 cells (Figure 8A, upper right panel).

To investigate the contribution of host immune surveillance to this phenotype, a similar experiment was performed using immunocompromised NOD SCIDγ mice. These lack mature B cells, T cells, natural killer cells, and have defective macrophages and dendritic cells. In this experiment, all of the mice in the three groups developed tumors within a comparable time period (Figure 8A, lower left panel) and only the mice injected with the A20 Foxp1 E7 clone showed a modest improvement in overall survival (Figure 8A, lower right panel). In A20 tumors harvested from both experiments immunohistochemical staining confirmed the loss of tumor cell Foxp1 protein expression *in vivo* in the CRISPR clones (Figure 8B).

## Discussion

The syngeneic A20 B-cell line grown in BALB/c mice has been previously characterized as a high-fidelity immunocompetent disseminated lymphoma model that recapitulates many aspects of human DLBCL (11). This model thus provides a system in which to study the interplay between a B-cell lymphoma and its immune microenvironment. Importantly, FOXP1 is a marker of adverse outcome in other B-cell malignancies, including marginal zone lymphoma (25), chronic lymphocytic leukemia (26), and follicular lymphoma (27, 28), Foxp1 expression patterns (both full length protein and smaller isoforms) and its repression of MHC-II, previously observed in human ABC-DLBCL (9), are both conserved in the murine A20 model. The current study also demonstrates that transient Foxp1 silencing in the A20 cell line did not compromise cell viability. While FOXP1 depletion is commonly toxic for human DLBCL cell lines (10), multiple studies have reported that viability and proliferation of the human OCI-Ly10 ABC-DLBCL cell line are also unaffected by FOXP1 depletion (3, 29). The A20 model therefore represents a relevant biological system for further investigating the role of Foxp1 in immune surveillance *in vivo*.

In the absence of any *in vitro* loss of viability on Foxp1 silencing in A20 cells, CRISPR/Cas9 genome editing was selected as a genetic approach to constitutively deplete Foxp1 expression. Expression of the full length Foxp1 protein was effectively silenced using all three CRISPR gRNAs, whilst only those targeting exons 6 and 7 also disrupted expression of the smaller Foxp1 isoforms; exon 6 targeting eliminated the highest molecular weight smaller isoform while exon 7 targeting eliminated all of the Foxp1 isoforms seen in the parental A20 cells. This exon 6 targeting data also demonstrates, for the first time, that the smaller abundant Foxp1 protein isoforms, in A20, are encoded by distinct transcripts.

Further *in vitro* characterization of A20 clones targeting either *Foxp1* exon 6 or 7, confirmed upregulation of cell surface MHC-II expression. However, upregulation of Cd74 expression (seen in human DLBCL cell lines), which is normally coregulated with MHC-II by the class II major histocompatibility complex transactivator (CIITA), was not consistently observed. RNA sequencing analysis identified a set of downregulated genes that were common to both the *Foxp1* exon 6- and 7-targeted A20 CRISPR clones. MHC-II was not represented among these genes and thus it is likely that post-transcriptional regulation may be important in A20 cells. This would be consistent with the lack of co-regulation of Cd74, which is normally mediated transcriptionally by CIITA. Interestingly, the G protein-coupled receptor Lgr6 is an epithelial stem cell marker that has been shown to promote Wnt receptor signaling and drive progression in multiple cancer subtypes (23). Lgr6 activation by Foxp1, in the current study, is consistent with FOXP1 being identified as an activator of Wnt signaling in human DLBCL (30), raising the possibility that this pathway may also be important in A20 cells. Silencing the full-length FOXP1 protein had previously been shown to inhibit CD19 expression in human DLBCL cell lines (15), and Cd19 expression at both the transcript and protein level was dramatically reduced in the A20 Foxp1 CRISPR clones. Cd19 is a B-cell marker and an important therapeutic target in B-cell malignancies. Importantly, it defines the intrinsic and antigen receptor-induced signaling thresholds that regulate clonal B-cell expansion and humoral immunity (31). CD19 cross-linking has also been reported to inhibit B-cell receptor-mediated MHC-II antigen processing and presentation (32). Thus driving Cd19 expression potentially provides a further mechanism by which Foxp1 could suppress antigen presentation by the remaining MHC-II molecules. In addition to presenting antigens to T cells, MHC-II also has a signal transducing role that regulates B-cell function and which can be enhanced by CD19 (33). For example CD19 has been demonstrated to be required for normal MHC class II-mediated signaling, including sustained Akt activation and proliferation in primary B cells (34). However, the relevance of this functionally cooperative signaling role in A20 cells in unclear, as Cd19 and MHC-II are differentially regulated by Foxp1.

The Foxp1 A20 CRISPR clones also share common hallmark signatures, including DNA repair, oxidative phosphorylation, and MYC targets, which are implicated in the pathogenesis of human B-cell malignances. FOXP1 expression has been reported to be upregulated during high-grade B-cell lymphoma transformation by MYC-mediated repression of the microRNAs *miR-34a* and *miR-150* (28, 35). FOXP1 itself also directly activates *c-MYC* in human ABC-DLBCL (3), thus providing a feedback loop that reinforces this oncogenic pathway. *miR-34* also downregulates FOXP1 expression during the p53/DNA damage response in B-cell lymphocytic leukemia, to limit their B-cell receptor signaling (36). It is also notable that MYC-driven DLBCL (where FOXP1 is commonly upregulated as a consequence of MYC suppression of *miR-34a*) are sensitive to inhibitors of the DNA damage response, which is being explored as a therapeutic strategy (37).

In addition to classification based on their cell-of-origin, molecular profiling of human DLBCL has also identified subtypes characterized by other processes, including oxidative phosphorylation (38). The FOXO1 forkhead transcription factor has been identified as the major sensor and effector of oxidative stress in the Ox-Phos-DLBCL subtype (39). FOXP1 and FOXO1 already have an established relationship, for example Foxp1 antagonizes the induction of *IL7RA* by Foxo1 in naïve T cells via competition for binding to its enhancer (40). In some cell types, FOXP1 also drives a negative feedback loop to suppress FOXO-induced apoptosis (41); although the absence of a viability phenotype suggests this is not the case in A20 cells. Thus, in the future it will be interesting to investigate whether the functional interplay between Foxp1 and Foxo1 might contribute to the regulation of oxidative phosphorylation in A20 and in human DLBCL. Oxidative phosphorylation generates toxic reactive oxygen species and its targeting is actively being explored as a therapeutic strategy in DLBCL. A durable partial response to OPB-111077, a small-molecule STAT3 and oxidative phosphorylation inhibitor, has already been observed in a DLBCL patient in a recent Phase I study in advanced cancers (42).

In the current study we have demonstrated that immune suppression is a critical mechanism by which tumor cell expression of Foxp1 promotes *in vivo* growth in the A20 B-cell lymphoma model. The improved survival of NOD SCIDγ mice bearing the Foxp1 exon 7 A20 clone may reflect the more complete ablation of Foxp1 expression and is consistent with the *in vitro* ability of Foxp1 to modulate multiple pathways with an important role in lymphoma pathogenesis, not just immune surveillance (3, 9). Upregulation of MHC-II improves the presentation of lymphoma antigens, making T cells a likely candidate for contributing to the Foxp1 depletion phenotype. This is also consistent with T-cell activation signatures being affected by FOXP1 silencing in human DLBCL cell lines (9). It is also possible that Foxp1 depletion may improve lymphocyte migration into the tumor microenvironment, as is the case in breast cancer where FOXP1 has recently been identified as a negative regulator of tumor infiltrating lymphocyte migration (43). However, preliminary *ex vivo* immunohistochemical analysis of harvested A20 tumors only found reduced numbers of CD3^+^ and CD8^+^ T cells in the Foxp1 exon 7 targeted tumors (data not shown), suggesting that reduced T-cell infiltration may not explain the common phenotype shared by both A20 clones.

There is considerable interest in identifying biomarkers for predicting the response of cancer patients to immune checkpoint inhibitors and for identifying synergistic combination therapies including these agents. The expression levels of PD-1 on tumor infiltrating lymphocytes and tumor cell expression of PD-L1 and/or PD-L2 have been correlated with adverse clinical outcome in multiple studies of human DLBCL (44). Nivolumab (anti-PD-1) exhibited modest efficacy as a monotherapy in relapsed DLBCL, with an overall response rate of 36% (45). More promisingly, 51% of DLBCL patients achieved an overall response when treated with Pidilizumab (anti-PD-1) as a consolidation strategy after autologous stem cell transplantation (46). Interestingly, FOXP1 and MHC-II expression have both been identified, in independent studies, as markers that predict the response of patients with solid tumors to immune checkpoint inhibition. In non-small-cell lung cancer, an unmethylated *FOXP1* locus was predictive of improved progression-free and overall survival in patients treated with anti-PD-1 (47). While this initially seems counterintuitive to the data presented here, FOXP1 can act as either an oncogene or a tumor suppressor depending on the cellular context and, thus, this does not exclude the potential for FOXP1 expression to predict a poor response to immune checkpoint inhibition in DLBCL patients. In melanoma patients tumoral MHC-II expression has also been associated with a favorable response to anti-PD-1 therapy and has been proposed as a biomarker for predicting patient response (48, 49). It will be interesting in future to assess both FOXP1 and MHC-II expression for their ability to predict the response of DLBCL patients to immune checkpoint inhibitors.

In summary we have developed an *in vivo* A29 lymphoma model in which stable Foxp1 depletion by CRISPR/Cas9 genome editing delayed *in vivo* lymphoma growth and appeared to be curative in a subgroup of animals. Further studies are needed to comprehensively explore the immunological mechanisms by which Foxp1 impairs lymphoma growth. Future cell depletion experiments in an immunocompetent host will enable identification of the immune cell types that mediate this immune surveillance and then the mechanisms by which they do so can be elucidated. This model will facilitate further characterization of the mechanisms by which tumoral Foxp1 depletion can restore immune surveillance and offers an opportunity to investigate the potential of this approach to enhance the response to immune checkpoint inhibitors in DLBCL.

## Data Availability Statement

The datasets generated for this study can be found in GSE139536 (https://www.ncbi.nlm.nih.gov/geo/query/acc.cgi?acc=GSE139536).

## Ethics Statement

The animal study was reviewed and approved by the Medical Sciences Animal Ethics Committee, University of Oxford.

## Author Contributions

SF and AB conceived and designed the study and drafted the manuscript. SF, AA, SM, DG, RA, KW, DL, and AB contributed to acquisition of data, and/or analysis, and interpretation of data. All authors contributed to the revision and approval of the final version of the manuscript.

### Conflict of Interest

The authors declare that the research was conducted in the absence of any commercial or financial relationships that could be construed as a potential conflict of interest.
