# Supplementary material for: CRISPR/Cas9-Mediated Foxp1 Silencing Restores Immune Surveillance in an Immunocompetent A20 Lymphoma Model
Source: Front Oncol. 2020 Apr 3;10:448. doi: 10.3389/fonc.2020.00448 (PMC7145990; doi:10.3389/fonc.2020.00448)
Supplement: Supplementary file 1 [file Data_Sheet_1.pdf]

SUPPLEMENTARY INFORMATION

Supplementary Table 1. List of siRNA sequences used for transient *Foxp1* silencing (Life Technologies)

siRNA ID	Sequence (sense)	Sequence (antisense)
**MSS246912**	GGUUCACACGAAUGUUUGCUUACUA	AAGUAAGCAAACAUUCGUGUGAACC
**MSS246913**	CCAUGACAGAGAUUACGAAGACGAA	UUCGUCUUCGUAAUCUCUGUCAUGG

Note: siRNA MSS246912 is designated si*Foxp1* #1 in the text and figures, siRNA MSS246913 is designated si*Foxp1* #2.

Supplementary Table 2. List of antibodies used for Western blotting, flow cytometry and immunohistochemistry.

Antibody	Isotype	Use	Supplier
**Mouse & human FOXP1**	Mouse IgG2a	Western blot (1:30)	Banham lab (University of Oxford) Clone JC12
**Mouse MHC Class II (I-A) conjugated to PE**	Rat IgG2b	Flow cytometry (1:500)	eBioscience 12-5322-81 clone NIMR-4
**Mouse Cd74 conjugated to APC**	Rat IgG1	Flow cytometry (0.1 ug + 5 ul mouse serum)	R&D Systems FAB7478A
**Mouse & human FOXP1**	Rabbit IgG	Western blot (1:2000), IHC (1:100)	Cell Signaling Technology D35D10
**Nucleophosmin (Npm)**	Mouse IgG1	Western blot (1:1000)	Banham lab (University of Oxford) Clone NA24
**PE Isotype control**	Rat IgG2b	Flow cytometry (1:500)	eBioscience 12-4031-82 clone eB149/10H5
**PE Isotype control**	Rat IgG2a	Flow Cytometry (10ug/ml)	eBioscience 12-4321-81 Clone eBR2a
**APC isotype control**	Rat IgG1	Flow cytometry (1:500)	eBioscience 17-4301-82 Clone eBRG1
**Mouse Cd19 conjugated to PE**	Rat IgG2a	Flow cytometry (10ug/ml)	eBioscience 12-0193-81 Clone eBio1D3
**Beta-actin (mammalian)**	Mouse IgG1	Western blot (1:20 000)	Sigma-Aldrich AC-15

Abbreviations: APC: allophycocyanin; PE: phycoerythrin.

Supplementary Table 3. Primers for SYBR Green qPCR

Gene	Forward primer sequence (5'-3')	Reverse primer sequence (5'-3')
Cd19	CCTGGGCATCTTGCTAGTGA	CCGGAACATCTCCCCACTAT
Phyhip	ACACAGAGTCTCTCCCTTCC	TGGTTTGGCTCCTGGCT
Iglv2	TTCCCAGGCTGTTGTGACTC	ACTAGTTGTAACAGCCCCAGT
Lgr6	GAGTTGGAATGCCATCCGTG	AGTCAGGTCCAGCTTAACCAA
Hprt	TGGGCTTACCTCACTGCTTT	TCATCGCTAATCACGACGC
B2m	TCACACTGAATTCACCCCCA	TCTCGATCCCAGTAGACGGT

Supplementary Table 4. Taqman primers and probes (Life Technologies)

Taqman assay ID	Gene name	Fluorophore
**Mm00474854_m1**	*Foxp1* (forkhead box P1)	FAM-MGB
**4318839**	*18S* (18S ribosomal RNA)	VIC

Supplementary Table 5. Guide sequences for CRISPR/Cas9 genome editing, targeting murine *Foxp1* (Sigma-Aldrich).

***Foxp1* exon targeted**	**Strand**	**Sequence**
**Exon 5**	Top strand	**CACCG**GTGGATTAAAGTCTCCCAAG
**Exon 5**	Bottom strand	**AAAC**CTTGGGAGACTTTAATCCAC**C**
**Exon 6**	Top strand	**CACCG**AGTGGCTATGATGACACCTC
**Exon 6**	Bottom strand	**AAAC**GAGGTGTCATCATAGCCACT**C**
**Exon 7**	Top strand	**CACCG**CTTCTCCAACAGCAACATGC
**Exon 7**	Bottom strand	**AAAC**GCATGTTGCTGTTGGAGAAG**C**

Supplementary Table 6. List of primer sequences used for sequencing CRISPR DNA plasmid in bacterial colonies and minipreps (Sigma-Aldrich)

Forward primer used is specific to pX330 vector backbone (ampicillin resistance gene) and reverse primer is specific to guide sequence.

Primer name	Direction	Sequence
**Fwd PX330 Amp**	Forward	AACGTTTTCCAATGATGAGC
***Foxp1* E5 bottom strand**	Reverse	**C**CACCTAATTTCAGAGGGTTC**CAAA**
***Foxp1* E6 bottom strand**	Reverse	**C**TCACCGATACTACTGTGGAG**CAAA**
***Foxp1* E7 bottom strand**	Reverse	**C**GAAGAGGTTGTCGTTGTACG**CAAA**

Supplementary Table 7. Differentially expressed genes (Excel file)

Differentially expressed genes (FDR adjusted *p*-value, $p<0.05$) in each of the four *Foxp1* CRISPR clones (down or upregulated) with $\log_2$ fold change values (LFC). These lists were used to create the Venn diagrams in Figure 4A.

Supplementary Table 8. Differentially expressed genes in Hallmark pathways (Excel file)

Differentially expressed genes found in Hallmark pathways from gene set enrichment analysis. Genes are ordered by $\log_2$ fold change (LFC).
